# Single Oral Dose Pharmacokinetics of Decursin and Decursinol Angelate in Healthy Adult Men and Women

**DOI:** 10.1371/journal.pone.0114992

**Published:** 2015-02-19

**Authors:** Jinhui Zhang, Li Li, Thomas W. Hale, Wayne Chee, Chengguo Xing, Cheng Jiang, Junxuan Lü

**Affiliations:** 1 Department of Biomedical Sciences, Texas Tech University Health Sciences Center School of Pharmacy, Amarillo, Texas, United States of America; 2 Clinical Research Unit, School of Medicine, Texas Tech University Health Sciences Center, Amarillo, Texas, United States of America; 3 Department of Medicinal Chemistry, College of Pharmacy, University of Minnesota, Minneapolis, Minnesota, United States of America; University of Colorado Denver, UNITED STATES

## Abstract

The ethanol extract of *Angelica gigas* Nakai (AGN) root has promising anti-cancer and other bioactivities in rodent models. It is currently believed that the pyranocoumarin isomers decursin (D) and decursinol angelate (DA) contribute to these activities. We and others have documented that D and DA were rapidly converted to decursinol (DOH) in rodents. However, our *in vitro* metabolism studies suggested that D and DA might be metabolized differently in humans. To test this hypothesis and address a key question for human translatability of animal model studies of D and DA or AGN extract, we conducted a single oral dose human pharmacokinetic study of D and DA delivered through an AGN-based dietary supplement Cogni.Q (purchased from Quality of Life Labs, Purchase, NY) in twenty healthy subjects, i.e., 10 men and 10 women, each consuming 119 mg D and 77 mg DA from 4 vegicaps. Analyses of plasma samples using UHPLC-MS/MS showed mean time to peak concentration (*T_max_*) of 2.1, 2.4 and 3.3 h and mean peak concentration (*C_max_*) of 5.3, 48.1 and 2,480 nmol/L for D, DA and DOH, respectively. The terminal elimination half-life (*t_1/2_*) for D and DA was similar (17.4 and 19.3 h) and each was much longer than that of DOH (7.4 h). The mean area under the curve (AUC_0-48h_) for D, DA and DOH was estimated as 37, 335 and 27,579 h∙nmol/L, respectively. Gender-wise, men absorbed the parent compounds faster and took shorter time to reach DOH peak concentration. The human data supported an extensive conversion of D and DA to DOH, even though they metabolized DA slightly slower than rodents. Therefore, the data generated in rodent models concerning anti-cancer efficacy, safety, tissue distribution and pharmacodynamic biomarkers will likely be relevant for human translation.

**Trial Registration:**

ClinicalTrials.gov NCT02114957

## Introduction

**Fig 1:**
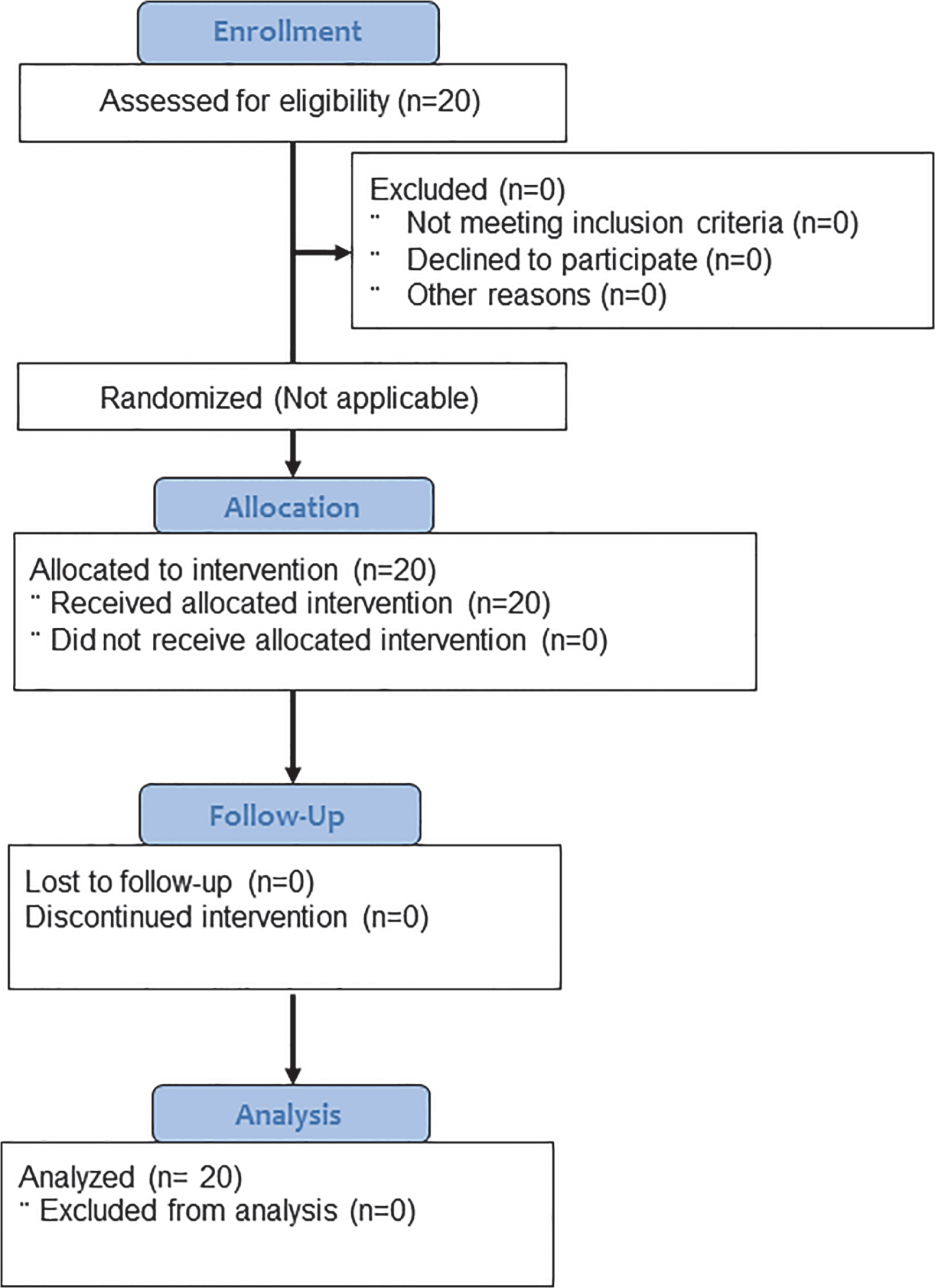
CONSORT flow diagram for single dose PK study of Cogni.Q pyranocoumarins.

**Fig 2:**
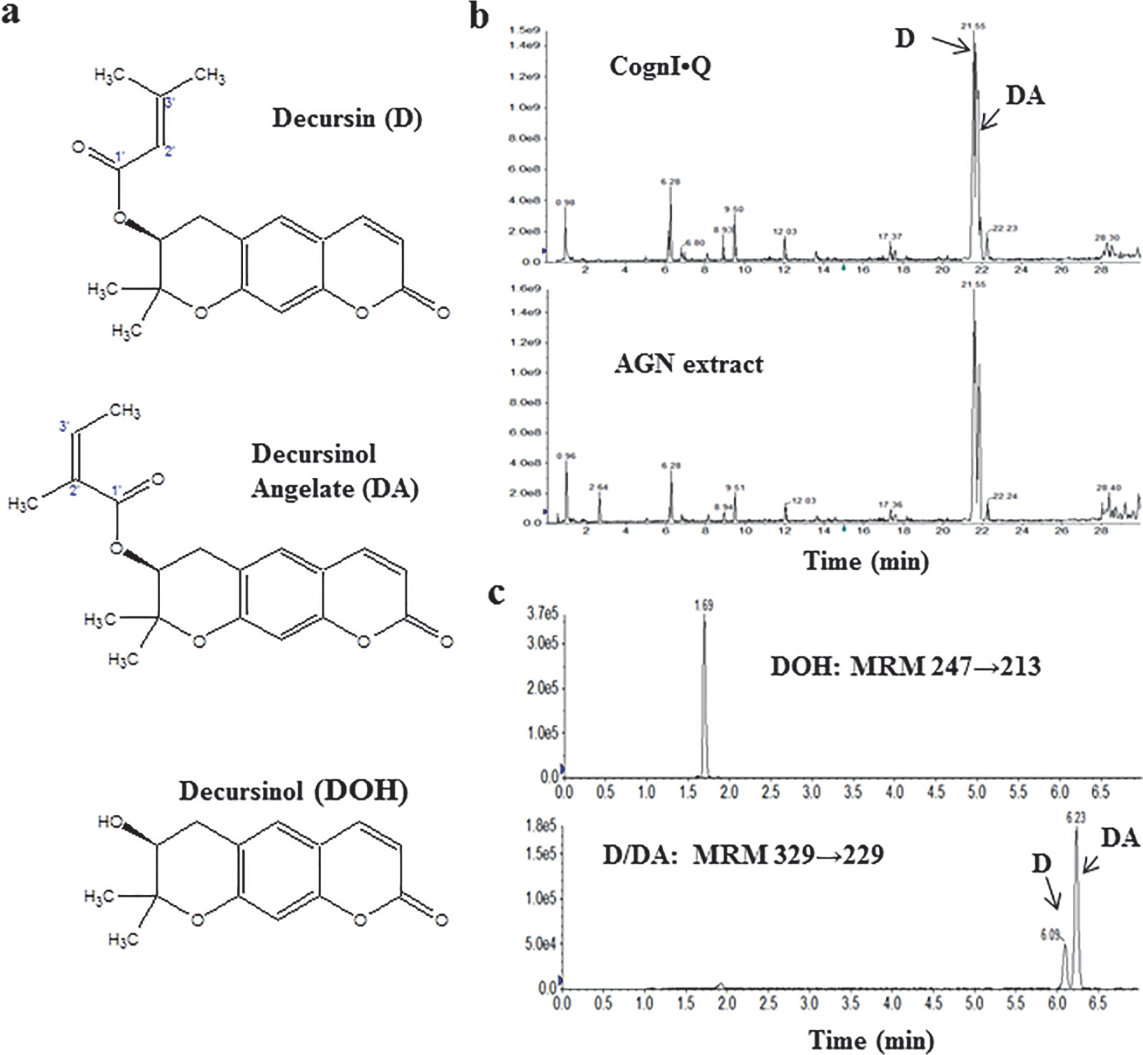
Chemical structures of pyranocoumarins, UHPLC characterization of test material Cogni.Q and example of UHPLC analysis of a plasma sample. a) Structure of decursin (D), decursinol angelate (DA) and decursinol (DOH); b) Chemical fingerprinting of Cogni.Q and AGN extract by UHPLC-MS/MS in full scan mode (EMS scan); c) Typical chromatograms of D, DA and DOH in human plasma by UHPLC-MS/MS in MRM mode.

We report here a human pharmacokinetic (PK) study (See Fig. 1 CONSORT flow diagram) of pyranocoumarin compounds administered through a dietary supplement of *Angelica gigas* Nakai (AGN). AGN is a traditional medicinal herb widely used in Korea and some Asian countries [1]. AGN-containing products (*e.g.*, Cogni.Q studied in the current study, Ache Action, Decursinol-50, Fast Acting Joint Formula, EstroG-100/Profemin) are marketed as dietary supplements for pain relief, memory improvement and women’s health in the United States. Decursin (D) and its isomer decursinol angelate (DA) (Fig. 2A) are the major chemical components in the ethanol extract of the root of AGN [2]. The *in vitro* and *in vivo* anti-cancer, neuro-protective and other biological activities of D and DA as well as AGN extract, have been reported in the past decade (see our comprehensive review [2] and other references [3–6]). The *in vivo* anti-cancer efficacy of AGN has been reported in prostate [3] [7] [8] and lung [8]. The dosages of AGN used in these models ranged from 100–200mg/kg, equivalent to 20–100mg/kg D/DA depending on extraction procedures. Some AGN extract-based dietary supplements have been studied for their activities against Alzheimer’s type dementia in clinical trials in Korea [9,10]. The 3-herbal mixture EstroG-100 (AGN, *Cynanchum wilfordii, Phlomis umbrosa*) has been found beneficial to relieve many post-menopausal symptoms in US women [11].

Our long-term goal is to develop effective AGN modalities or compounds for the prevention, treatment and/or palliative care of cancers of the prostate and other organs. Currently it is believed that D and DA mediate most of the reported anti-cancer activities of AGN extract in cell culture models [2]. We and others have in *in vitro* studies demonstrated direct anti-cancer activities of D and DA, as evident by induction of apoptosis and cell cycle arrest, against prostate [12–14] and breast cancer cells [15]. In rodent models, we and others have shown that both D and DA are rapidly converted to decursinol (DOH) (Fig. 2A) in rats and mice after oral gavage or intraperitoneal (*i.p.*) injection, either in the forms of pure chemicals or ethanol extracts [8,16–18]. In sharp contrast to D and DA, up to 100 μM of DOH had little anti-proliferative effect on cultured prostate and breast cancer cells [12,13,15].

Regarding the likely organ site(s) for conversion of D and DA to DOH, previous work by us and others indicated the liver, instead of the gastrointestinal tract and blood, might be the primary organ in rodents [17,18]. Since it is known that interspecies differences in oral bioavailability typically arise as a result of differences in first pass metabolism in the liver, we used liver S9 fraction from humans and rodents to compare the *in vitro* metabolism of D and DA [19]. Such *in vitro* data showed that D and DA were metabolized more slowly by human liver S9 fraction than by mouse and rat liver S9 fraction [19] and led us to hypothesize that, after oral administration, humans and rodents might metabolize D and/or DA differently in either a qualitative or quantitative manner.

Considering the notable differences in the biological activities of D and DA *vs*. their major metabolite DOH in the cell culture models and the S9 data suggesting a potential human *vs*. rodent metabolic difference toward D and DA, we reasoned that a comparison of their PK parameters in humans with rodent models would be crucial and timely to address the questions of human translational applicability of bio-efficacy data and mechanism extrapolation from rodent models. To this end, we conducted a study in 20 healthy human subjects, evaluating the single-dose PK of D and DA after oral delivery of AGN-containing dietary supplement Cogni.Q (purchased from Quality of Life Labs, Purchase, NY). We also compared their PK parameters among men and women.

## Materials and Methods

The IRB protocol A12-3742 for the human study was approved by Texas Tech University Health Sciences Center (TTUHSC) Institutional Review Board on 01/13/2013. The IRB protocol for this trial and supporting TREND checklist are available as supporting information; see S1 TREND Checklist and S1 Protocol. This trial had been registered in Clinicaltrials.gov with Identifier NCT02114957 (https://clinicaltrials.gov/).

### Subject selection

Ten healthy male and 10 female subjects were recruited by public announcement in Amarillo, TX, from January 2013 to April 2013 (see Fig. 1 CONSORT diagram). The sample size for each gender was chosen without power calculation due to the lack of prior knowledge of PK parameter variance in humans. After receiving informed consent verbally and in writing, the health status of each candidate was confirmed by health questionnaire, plasma biochemistry (hepatic and renal functions), complete blood cell count (CBC), and a physical examination by a participating academic physician. Subjects with diabetes, or pregnant, perinatal and breastfeeding women were excluded. Pre-dose characteristics of the subjects are summarized in Table 1. For the 48 hours preceding study visit until the final blood draw, all subjects were instructed to avoid taking any prescription or nonprescription drugs, vitamins, and herbal/dietary supplements. Each subject was compensated for participating in the study per TTUHSC IRB approved rate.

**Table 1:** Baseline characteristics of healthy subjects and dosage information for decursin (D) and decursinol angelate (DA) normalized to body weight.

	**All n = 20**	**Men n = 10**	**Women n = 10**	**Men vs. women unpaired t-test, 2-sided p**
Age in years, Mean (SD)	33.5 (10)	31.3 (10.5)	35.6 (9.3)	0.344
Race, Caucasian, # (%)	13 (65%)	6 (60%)	7 (70%)	
Hispanic, # (%)	4 (20%)	2 (20%)	2 (20%)	
Asian, # (%)	3 (15%)	2 (20%)	1 (10%)	
Body Weight in kg, Mean (SD)	76.4 (11.1)	82.7 (6.5)	71.0 (12.4)	0.027
D dose, mg/kg, Mean (SD)	1.60 (0.27)	1.47 (0.12)	1.74 (0.33)	0.036
DA dose, mg/kg, Mean (SD)	1.03 (0.18)	0.94 (0.07)	1.11 (0.21)	0.036
Total D/DA dose, mg/kg, Mean (SD)	2.63 (0.45)	2.41 (0.19)	2.85 (0.54)	0.036

### Investigational herbal product

Because neither D nor DA had been approved as Investigational New Drugs (INDs) in the United States, we selected the commercially available dietary supplement Cogni.Q which contained patented AGN ethanol extract “INM-176” for this study. We obtained written confirmation from U.S. Food and Drug Administration Center for Drug Evaluation and Research (CDER/FDA) that no IND approval was required for using this dietary supplement for the proposed PK study.

Using an UHPLC system (Nexera, Shimadzu Corporation, Columbia, MD, U.S.A) combined with a hybrid triple quadruple linear ion trap mass spectrometer (QTRAP 5500, AB Sciex, Framingham, MA, USA), we compared the chemical fingerprints of Cogni.Q content and the AGN extract that we routinely used for animal studies. Chromatographic separation was performed on a Kinetex C18 column (100mm×3mm×2.6μm, Phenomenex, Torrance, CA, USA). The column oven temperature was kept at 40°C. The mobile phase was water (solvent A) and acetonitrile (solvent B) in gradient elution. The initial mobile phase was set at 10% B followed by a gradient to 62% B in 25 min, and then to 95% B in 2 min. After that, the mobile phase was kept at 95% B for 3 min. The flow rate was set 0.4 mL/min. An Information-dependent acquisition (IDA) method was utilized in which EMS (full scan mode with linear ion trap) functioned as a survey scan, and EPI (Enhanced Product Ion) Scan was triggered for MS/MS scans by IDA criteria. The ionization was performed in positive ESI mode, source parameters were curtain gas 30, CAD gas high, ion spray voltage 5500 V, source temperature 575°C, Gas1 at 50psi, Gas2 60psi. EMS scan was performed from 150 to 500 Da, scan rate 10000 Da/s, with dynamic fill time on, declustering potential (DP) 100, EP (entrance potential) 10 and CE (collision energy) 10. According to IDA criteria, two most intense ions with intensity above 105 counts per second detected by EMS were selected for further EPI scans. EPI scans were performed at scan range from 80 to 500 Da, scan rate 10000 Da/s, CE 35 with CES (collision energy spread) 15. The data were collected and processed by Analyst 1.5.2. As can be seen in Fig. 2B, the high degree of similarities in the profiles with respect to peaks and their relative contents confirmed that Cogni.Q contained AGN extract with pyranocoumarin profiles nearly identical to the AGN extract evaluated in our rodent models. The suggested daily dosage for adults of 4 vegicaps (119 ± 9 mg D and 77 ± 5 mg DA per our HPLC-UV analysis) was used.

### Treatment plan and bio-specimen collection

Eligible subjects arrived at our clinical facility at 0700 (7:00 am) on the test day after a fast beginning at 2200 (10:00 pm) the previous night. After a brief review of vital signs, the nurse placed an intravenous (IV) line with saline drip in one arm of each subject. A baseline pre-dose (hour 0) blood sample was drawn through the IV line; after that subjects swallowed four Cogni.Q vegicaps with 240 mL of water at hour 0 under direct supervision. Subjects stayed at the clinical facility in their room for the next 12 hours, returning at 24 and 48 hours. Blood was drawn at baseline (hour 0) and at 0.5, 1, 2, 3, 4, 6, 8, 12, 24 and 48 hours after dosing into Vacutainer Blood Collection Tube (Becton-Dickinson, Franklin Lakes, NJ. Product Number: 367874, Sodium heparin was used as anti-coagulant). Water was allowed as desired except for within the first hour after Cogni.Q consumption. All subjects received standardized meals at post-dose hours 4.5 and 9.5 but no food was allowed for at least the first 4 hours post-dose.

Plasma was prepared by centrifuging the heparinized blood at 1000 g for 15 minutes at room temperature. Urine samples (40mL) were collected within time spans of 0–4, 4–8, 8–12 and 12–24 hours (total volume of urine was recorded). All plasma and urine samples were put on dry ice immediately and then stored at -80°C freezer until analysis.

### Safety evaluation

Vital signs were taken and recorded pre-dose hour 0 and post-dose hours 1, 5, 8, 12, 24 and 48. Additional blood samples collected at hours 0 (baseline) and 24 were tested for hepatic function panel, blood urea nitrogen (BUN) & creatinine panel (renal function) and CBC by a College of American Pathologists (CAP) certified clinical laboratory (Quest Diagnostics, Dallas, TX). On the 30th day post-dose, all participants were contacted by telephone for follow-up adverse event assessments.

### Analytical chemicals and reagents

Decursin and DA were first co-purified as a mixture from an ethyl acetate soluble fraction of the AGN extract by silica chromatography [16,17]. The D/DA mixture was further purified using the HPLC system to separate D and DA as described before [19]. DOH was prepared by hydrolysis of D/DA mixture as reported previously [17]. The purity of D, DA and DOH was verified to be higher than 99% by HPLC. Prednisolone (>99%, internal standard [IS] for UHPLC-MS/MS) and ethyl acetate (99.9%) were purchased from Sigma-Aldrich Co. (St. Louis, MO). HPLC grade methanol, acetonitrile and *tert*-butyl methyl ether were from Fisher Scientific (Pittsburgh, PA). B&J brand LC-MS grade water and acetonitrile were from Honeywell (Morristown, NJ).

### Extraction of plasma and urine samples for UHPLC-MS/MS analyses

Plasma samples were extracted with a validated solid supported liquid extraction (SLE) method facilitated with a positive pressure manifold (Biotage, Charlotte, NC). Briefly, 100 μL of plasma sample was spiked with 5 μL of 10 ng/μL prednisolone (IS), diluted with 200 μL of LC-MS grade water, mixed and loaded onto ISOLUTE SLE+ 400 μL Array Well cartridges (Biotage, Charlotte, NC). A pulse of minimum positive pressure was applied to facilitate the samples absorption into the cartridges. After the samples were allowed to absorb for 5 min, the compounds were eluted with 2×500 μL *tert*-butyl methyl ether. The eluent was dried in a Speedvac and the resulting residue was reconstituted in 50 μL of methanol for UHPLC-MS/MS analysis.

Urine samples from each subject collected from 0 to 24 hours post-dose were pooled proportional to the volume of urine in each sampling period. Urine sample (200μL) was spiked with 5 μL of prednisolone solution (10 ng/μL, IS), and extracted with 1500 μL of ethyl acetate. The ethyl acetate fraction was recovered and dried using a SpeedVac. The dried residue was reconstituted in 50 μL of methanol for UHPLC-MS/MS analysis and 5 μL of the sample was injected.

Eight-point calibration curves were constructed for D, DA and DOH, at concentrations of 0.1, 0.5, 1, 5, 10, 20, 50 and 100 μg/L for D and DA, and at concentrations of 1, 5, 10, 50, 100, 200, 500 and 1000 μg/L for DOH, respectively. The linear range was 0.1–100 μg/L (0.3–300 nmol/L), 0.1–100 μg/L (0.3–300 nmol/L) and 1–1000 μg/L (4–4000 nmol/L) for D, DA and DOH, respectively. The inter- and intra-assay CV were lower than 6.5% and the accuracy was between 90–105%. Calibration curve was run for each batch of samples analyzed. The mean calibration curve correlation coefficient (r) for D, DA and DOH was 0.999, 0.999 and 0.998, respectively (data from 7–10 calibration curves). In addition, 70% of the plasma samples were extracted and analyzed twice, the data from two replicate analyses were nearly identical and combined for final data analyses and presentation.

### UHPLC-MS/MS system and analytical method

The UHPLC-MS/MS system described above was used to quantitate D, DA and DOH [17,19]. The Analyst 1.5.2 software was used for data acquisition and the MultiQuant 2.2 was used for quantitative analysis of data.

Chromatographic separations were performed on tandem columns of a Kinetex XB-C18 column (100mm×2.1mm×1.7μm, Phenomenex) and a poroshell 120 EC-C18 column (50mm×3mm×2.7μm, Agilent). The column oven temperature was kept at 60°C. The mobile phase was water (solvent A) and acetonitrile (solvent B) as follows: 30% B followed by a gradient to 40% B in 0.5 min, and then to 58% B in 6.4 min. After that, a fast gradient to 90% B in 0.1 min was applied and then the mobile phase was kept at 90% B for 2 min. The flow rate was set at 0.6 mL/min. The injection volume was 5 μL. Under this condition, D and DA could be separated efficiently to the baseline (Fig. 2C).

The mass spectrometer was equipped with a TurboIonSpray ion source and was operated in a positive electrospray ionization (ESI) mode. Source parameters were optimized as follows: curtain gas 30 psi; collision gas high; ion spray voltage 5000 V; nebulizer gas (GS1) 60 psi; turbo gas (GS2) 60 psi; source temperature 550°C; EP 10; and dwell time 100 ms. The optimized multiple reaction monitoring (MRM) transitions and MS parameters of each analyte and IS are as follows: for D and DA, MRM transition used was at m/z 329→229 (Fig. 2C), DP 111, CE 29; for DOH, MRM transition at m/z 247→213 (Fig. 2C), DP 66 and CE 33; for prednisolone (IS), MRM transition m/z 361→171, DP 66 and CE 33.

### PK parameters and statistical analyses

The peak time (*T*_*max*_) and the peak concentration (*C*_*max*_) were determined directly from the individual plasma concentration-time curves without interpolation [20]. The area-under-the-plasma concentration versus time curve (AUC_0–48h_) was calculated using the Phoenix WinNonlin software Version 6.1 (Pharsight Corporation, Mountain View, CA) from plasma concentration versus time data.

The PK parameters were each analyzed by linear regression analyses against age or body weight to assess the magnitude of inter-subject variance accountable by these anthropometric data. Because of the precursor (D, DA) and product (DOH) relationship within each individual, one-tailed, paired-samples *t*-test was used to compare the means between pairs of *C*_*max*_, *T*_*max*_ and AUC_0–48h_ parameters within the subjects. For comparison between genders, two-tailed, unpaired-samples *t*-test was used for the group means of PK parameters, such as *C*_*max*_, *T*_*max*_ and AUC_0–48h,_ and terminal elimination half-life *t*_*1/2*_. When unequal variances were indicated by Levene’s test, Welch’s *t*-test was utilized instead. Effect size (Cohen’s *d*) and *post-hoc* power were estimated for each comparison per Cohen [21]. Statistical significance was determined at 0.05 alpha level. Statistical analyses were conducted using GraphPad Prism software (GraphPad Software, Inc. La Jolla, CA) and validated by R software [22].

## Results

### Stability of D and DA in fresh human whole blood and plasma

To rule out possible artifacts of blood collection, processing and blood-borne esterases, we tested the stability of D and DA in freshly obtained human whole blood and plasma. There was no loss of D and DA in plasma and whole blood after incubation at 37°C for 1 h, with no detectable generation of DOH and other metabolites (data not shown).

### Safety of dietary supplement Cogni.Q

The dietary supplement Cogni.Q was very well tolerated by all 20 subjects. No treatment related adverse effect such as fever, pain, nausea and rash occurred in any subject within first 48 hours. Comparison of plasma biochemistry and CBC results from blood collected at 0 and 24 hours showed no acute damage of liver, kidney and hematopoietic system associated with Cogni.Q consumption. At 30th day post-dose telephone follow-up, no participant reported an adverse event.

### Human plasma PK parameters for pyranocoumarins

**Fig 3:**
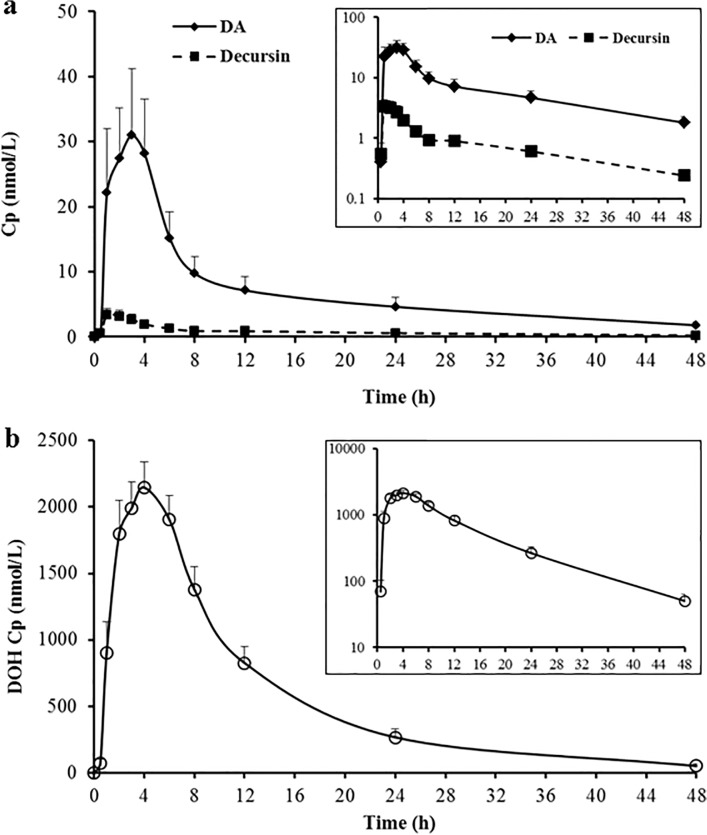
Human plasma concentration (Cp)-time profiles of pyranocoumarins after a single oral dose of Cogni.Q. (a) Parent compounds D and DA. (b) Product DOH. Mean ± SEM of all 20 subjects. Inserts show the semi-log plot of Cp versus time for corresponding analytes. Terminal *t*_*1/2*_ for D and DA was statistically the same (17.4 and 19.3 hours, respectively) and that for DOH was shorter (7.4 hours).

As shown in Fig. 3, DOH was the predominant pyranocoumarin metabolite in human plasma (Fig. 3B) whereas D and DA were detected at 2–3 orders of magnitude lower levels (Fig. 3A). The mean *C*_*max*_ for DOH, DA and D was 2480, 48 and 5.3 nmol/L, respectively (Table 2) and *C*_*max*_ for DA was greater than that for D (paired t-test, 1-sided, p = 0.001, post-hoc power 92%). The *T*_*max*_ for D and DA was each shorter than that for DOH (paired t-test, 1-sided, p<0.001, post-hoc power 80%; p = 0.002, post-hoc power 51%, respectively) (Table 2), consistent with their precursor-product relationship. Furthermore, *T*_*max*_ for D was statistically shorter than that for DA (paired t-test, 1-sided, p = 0.009). The mean AUC_0–48h_ values, reflecting cumulative plasma abundance of each analyte, were significantly different and in the order of D < DA << DOH (all p<0.01, Table 2), supporting >98% conversion of D/DA to DOH.

**Table 2:** Key pharmacokinetic parameters of decursin (D), decursinol angelate (DA) and decursinol (DOH) in all human subjects (n = 20) receiving dietary supplement Cogni.Q orally vs. those in rats.

**Analyte→PK parameter**	**D**	**DA**	**DOH**	**Statistical analyses**	**Linear coefficient of determination**
**Human**				P value (post-hoc power)	r^2^ values
***T*_max_, h, Mean (SD)**	2.1 (1.2)	2.4 (1.4)	3.3 (1.6)	Paired t-test, 1-sided	D vs. age r^2^ = 0.035
				D vs. DA p = 0.0094 (20%)	D vs. weight r^2^ = 0.0201
				D vs. DOH p = 0.0002 (80%)	DA vs. age r^2^ = 0.0014
				DA vs. DOH p = 0.0023 (51%)	DA vs. weight r^2^ = 0.0261
					DOH vs. age r^2^ = 0.096
					DOH vs. weight r^2^ = 0.071
***C*_max_, nmol/L Mean (SD)**	5.3 (4.7)	48.1 (56.4)	2480.3 (842.2)	Paired t-test, 1-sided	D vs. age r^2^ = 0.0492
				D vs. DA p = 0.0010 (92%)	D vs. weight r^2^ = 0.0214
				D vs. DOH p<0.0001 (>95%)	DA vs. age r^2^ = 0.0053
				DA vs. DOH p<0.0001 (>95%)	DA vs. weight r^2^ = 0.024
					DOH vs. age r^2^ = 0.0001
					DOH vs. weight r^2^ = 0.0004
**AUC_0–48h,_ nmol/L, Mean (SD)**	37.1 (29.2)	335.4 (398.0)	27579 (13769)	Paired t-test, 1-sided	D vs. age r^2^ = 0.164
				D vs. DA p = 0.0011 (92%)	D vs. weight r^2^ = 0.2178
				D vs. DOH p<0.0001 (>95%)	DA vs. age r^2^ = 0.009
				DA vs. DOH p<0.0001 (>95%)	DA vs. weight r^2^ = 0.059
					DOH vs. age r^2^ = 0.129
					DOH vs. weight r^2^ = 0.005
**Terminal *t*_*1/2*_, h, Mean (SD)** *	17.4 (6.8)	19.3 (8.5)	7.4 (2.0)	Paired t-test, 1-sided	D vs. age r^2^ = 0.0106
				D vs. DA p = 0.2406	D vs. weight r^2^ = 0.0186
				D vs. DOH p<0.0001 (95%)	DA vs. age r^2^<0.0001
				DA vs. DOH p<0.0001 (>95%)	DA vs. weight r^2^ = 0.0043
					DOH vs. age r^2^ = 0.2927
					DOH vs. weight r^2^ = 0.001
**Rat** ** **(n = 3)**					
***T***_**max**_, **h, median (range)**	1 (0.5–2)	1 (0.5–2)	4 (3–8)		
***C***_**max**_, **nmol/, Mean (SD)**	7.3 (4.0)	7.3 (3.4)	5638 (378)		
**AUC_0–48h_, h nmol/L, Mean (SD)**	36.0 (14.3)	64.3 (8.8)	81272 (6829)		

The human dose for D and DA was 1.6±0.3 and 1.0±0.2 (mean±SD) mg/kg, respectively.

*The terminal half-life values for four subjects were considered as outliers and were excluded.

**The rat data was from our previous publication [17]. By allometric scaling [27], the rat dose was 3.5 folds of the human dose.

Semi-log plot of plasma concentration versus time (Fig. 3A, inset) suggested that D and DA exhibited multiple compartmental characteristics. The substantial post-peak distribution phase decay of D and DA was likely related to their rapid liver destruction as well as extra-hepatic tissue uptake or re-distribution. The post-peak distribution phase for DOH was less dramatic as compared to the parent compounds. These results would be consistent with our earlier findings that the tissue/plasma ratios of D and DA were substantially greater than that of DOH in rodents [23]. Terminal elimination half-life *t*_*1/2*_ for D and DA was nearly identical (17.4 and 19.3 hours, respectively, p = 0.241) and each was much longer than that of DOH (7.4 hours, p<0.0001) (Table 2).

In spite of the extensive conversion of D and DA to DOH, humans retained more DA than D in plasma (Fig. 3A) (Table 2, *C*_*max*_ for DA vs. D, paired t-test, 1 sided p = 0.001). Even though each subject ingested roughly 50% more D than DA (119 vs. 77 mg) from the Cogni.Q vegicaps, the *AUC*_*0–48h*_ and *C*_*max*_ of DA were each almost 8 folds higher than those of D (Table 2) (paired t-test, one sided p = 0.001, post-hoc power 92% for both parameters). Similar trends have been observed in mice [16] and rats [17] in terms of the higher plasma level of DA vs. D. We recently reported that D and DA were converted to DOH by different enzyme systems in *in vitro* models: DA to DOH through oxidative hydrolysis catalyzed by cytochrome P450s exclusively, whereas D might be converted to DOH by both P450s and carboxylesterases [19]. This might explain why higher level of DA was present in plasma than D. The involvement of P450s in their conversion suggests pharmacological approaches with P450 inhibitor drugs to up-regulate their circulating and tissue contents.

Linear regression analyses of *C*_*max*_, *T*_*max*_, *AUC*_*0–48h*_ and terminal *t*_*1/2*_ for DOH with age showed coefficient of determination r^2^ values of 0.0001, 0.096, 0.129 and 0.2927, respectively and with body weight of 0.004, 0.071, 0.005, 0.001, respectively (Table 2). The r^2^ values for D or DA ranged from 0.0001 to 0.2178 (Table 2). Therefore, neither age nor body weight represented a major factor for the variance in these PK parameters.

### Gender effects on PK parameters

**Fig 4:**
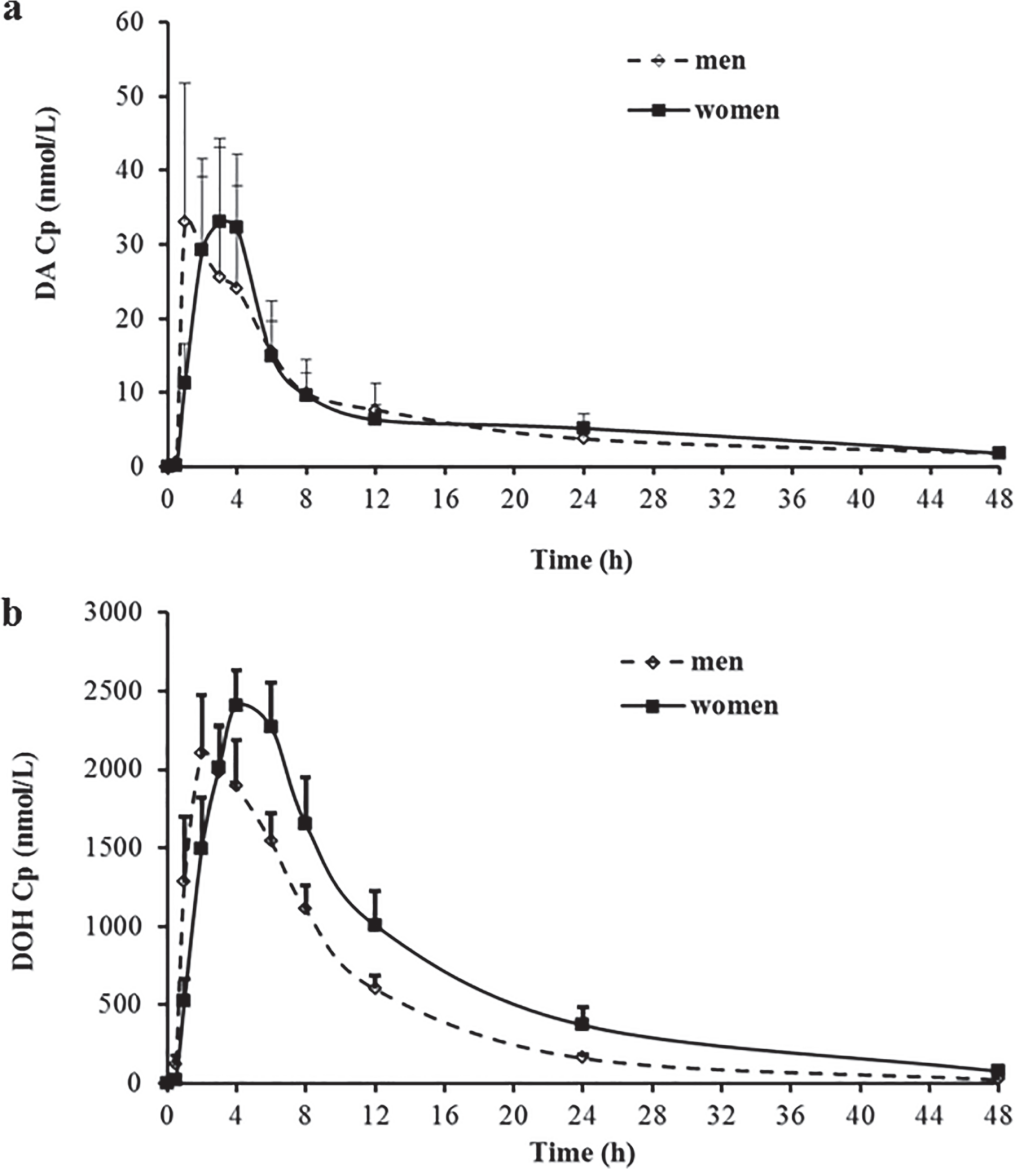
Gender comparison of plasma concentration (Cp)-time profiles of pyranocoumarins after a single oral dose of Cogni.Q. (a) DA. (b) DOH. Mean ± SEM. Men (n = 10) and women (n = 10).

The mean *C*_*max*_ values were not statistically different between men and women for D, DA or DOH (2-sided unpaired t-test) (Table 3). Nor were the AUC_0–48h_ for D, DA or DOH (unpaired t-test, 2-sided) (Fig. 4B, Table 3) statistically, although that for DOH approached threshold of α = 0.05 (p = 0.097, 2-sided t-test). The lighter body weight of the women than men (i.e., the men have a 15% higher average body weight than the women (Table 1) (unpaired t-test, 2 sided, p = 0.027) translated to the actual D and DA doses per unit body weight being 18% greater in women than that in men (unpaired t-test, 2-sided, p = 0.036). The DOH AUC_0–48h_ dose-corrected mean value of 27,713 for women was numerically closer to that of the men’s value of 22,457 (Table 3).

**Table 3:** Comparison of pharmacokinetic parameters of decursin (D), decursinol angelate (DA) and decursinol (DOH) in men vs. women receiving dietary supplement Cogni.Q orally.

**PK parameter**	**Analyte**	**Men N = 10**	**Women N = 10**	**Unpaired t-test, 2-sided, p**	**post-hoc power**
***T*_max_, h, Mean (SD)**	D	1.7 (1.2)	2.4 (1.1)	0.192	**72%**
	DA	2.1 (1.6)	2.7 (1.1)	0.335	
	DOH	2.4 (1.4)	4.1 (1.5)	**0.014**	
***C*_max_, nmol/L, Mean (SD)**	D	5.2 (5.1)	5.4 (4.9)	0.945	
	DA	52.2 (73.0)	43.9 (41.3)	0.760	
	DOH	2354 (950)	2606 (798)	0.529	
			2208 DCMV*		
***AUC*_*0–48h*,_ h.nmol/L Mean (SD)**	D	28.5 (20.8)	45.7 (34.7)	0.194	
	DA	327.3 (466.5)	343.6 (341.6)	0.930	
	DOH	22457 (8256)	32702 (16546)	0.097	
			27713 DCMV*		
***T*_*1/2*_, h, terminal Mean (SD)** **	D	17.7 (7.8)	17.0 (6.2)	0.851	**58%**
	DA	14.9 (2.8)	23.7 (10.1)	**0.044**	
	DOH	6.6 (1.9)	8.3 (1.7)	0.072	

*Dose-corrected mean value: due to lighter body weight of women in the study, their actual dose of D/DA was 1.18x higher than men on per kg basis (See Table 1).

**The terminal half-life values for four subjects were considered as outliers and were excluded (See S1 Table).

The mean *T*_*max*_ of D and DA (Fig. 4A) and DOH (Fig. 4B) in men were 1.7, 2.1 and 2.4 hour, whereas it was 2.4, 2.7 and 4.1 hour in women, respectively (Table 3). Although only the *T*_*max*_ of DOH was statistically different (unpaired t-test, 2-sided, p = 0.014, post-hoc power 72%), the above mentioned *T*_*max*_ numerical values for D and DA suggested faster absorption of these parent compounds in men than in women. Women are known to secrete less gastric acid than men (the basal gastric pH is approximately 0.5 unit greater than in men), and women also tend to have a more prolonged gastrointestinal transit time than men [24]. Though all subjects took the herbal product under fasting status, those inherent factors might have affected the dissolution of Cogni.Q vegicaps and absorption rate of D and DA, resulting in the slower *T*_*max*_ values of DOH for women (Table 3). For those biological activities that need repeated dosing such as for anti-cancer indications, the *T*_*max*_ difference between genders might not translate to substantial variation in efficacy. However, for an acute indication such as pain killing effects [25,26], in which the onset and duration of action need to be precisely controlled, the gender-difference should be considered for treatment regimen design. The terminal elimination half-life value (*t*_*1/2*_) for D or DOH was not different between men and women, whereas that for DA was statistically longer in women than in men (Table 3).

### Comparison with rodent PK parameters

By allometric scaling [27], the human dose of D (1.6 mg/kg) and DA (1.0 mg/kg) was approximately 28% of the dosage (*i.e.*, by a factor of 6) that we previously used in PK study in rats (33.5 and 21.5 mg/kg for D and DA, respectively) [17]. Considering the lower absolute and allometric dosages, the plasma *C*_*max*_ of DOH in humans was comparable to that of the rats. On the other hand, the *C*_*max*_ of DA was much higher in humans than rats (7 folds higher even without dose adjustment) (Table 2), indicating that humans metabolized DA much slower than rats. Nevertheless, DOH was still the dominant metabolite in the plasma of all 20 human subjects in spite of gender, age, and body weight. The average *C*_*max*_ of DOH was 50 folds higher than that of DA (Table 2). Therefore, the metabolic fate of D and DA in humans and rodents were qualitatively similar, with some minor differences at the level of the conversion rate of D *vs*. DA to DOH.

### Urinary excretion

Urine samples from each subject were pooled proportional to the amount of urine in each sampling period (hours 0–4, 4–8, 8–12 and 12–24). DA was detected in its original form in the urine of 14 subjects whereas D was only detected in the urine of 3 subjects. The levels of detectable D and DA ranged from 0.06∼1.74 nmol/L. DOH was detected in the urine of all subjects with a mean concentration of 289 nmol/L. These extractable unmodified pyranocoumarin forms accounted for the 24-hour cumulative excretion of less than 1% of the ingested pyranocoumarins. Further work is needed to estimate the extent of additional metabolites (free and conjugated forms) in urine and plasma.

## Discussion

To our knowledge, this is the first-in-human single oral dose PK study of D and DA in the United States. Consistent with AGN-based products being well tolerated in Korean populations according to a publication in a Korean journal [9], and online record in US Clinicaltrials.gov web site [10], we documented that AGN at the dosage tested in this study did not produce any observable adverse effect in the US subject cohort, particularly in Caucasians (65%, 13/20 in our cohort) and Hispanics (20%, 4/20 in our cohort). This information is important for future development of AGN modalities for the treatment and prevention of cancer and other diseases in the Americas and European countries.

Overall, our data demonstrated that the metabolic fate(s) of D and DA was qualitatively similar in humans and rodent models. Even though humans metabolized DA slower than rodents, both D and DA were still extensively converted to DOH (>98%). The information supports the extrapolatability of previous efficacy and toxicological evaluation data generated in rodent models [3,4,8,16,17,23,28]. In addition, the tissue distribution patterns of D/DA/DOH, and their *in vivo* pharmacodynamic biomarkers generated in rodent models will likely be relevant for human translation consideration of AGN products.

Scaled by simple allometry on the basis of body surface area [27], the human dose in this study was equivalent to a mouse dose of approximately 19 mg D/kg and 12 mg DA/kg (*i.e.*, by a factor of 12), within the dose range of previously reported *in vivo* anti-cancer efficacy in rodent models. For example, Son *et al* reported that the daily oral administration of D (10 mg/kg for 14 days) at 30 minutes prior to the injection of murine colon carcinoma CT-26 cells through tail vein reduced the formation of tumor nodules in the lungs and decreased lung weight caused by CT-26 metastases [29]. The plasma *C*_*max*_ of DOH in human subjects ranged from 1.3 to 4.0 μmol/L with an average value of 2.5 μmol/L (Table 2). At these concentrations, DOH has been reported to have biological activities in several cell culture models. For instance, Kang *et al* investigated the neuro-protective effects of DOH using primary cultures of rat cortical cells [25] and found that as low as 0.1 μmol/L exerted a strong protective effect on glutamate-injured cells. In another study investigating the anti-angiogenesis effect of DOH, Son *et al* reported that 1 μmol/L of DOH significantly inhibited the VEGF-induced proliferation and capillary-tube formation of human umbilical vein endothelial (HUVEC) cells [30]. We reported the effect of DOH on androgen receptor (AR) signaling in LNCaP cells, wherein 2–5 μmol/L of DOH decreased the secretion of prostate specific antigen (PSA) by 30–50% within 48 hours [13].

In this study, considerable variation was observed in human subjects for the plasma *C*_*max*_ of DA, with a mean of 48 nmol/L in 20 subjects and up to 200 nmol/L in 2 males (1 Caucasian and 1 Asian) (S1 Table, Individual data). We reported earlier that DA decreased secreted PSA levels in LNCaP cells with IC_50_ of 1 ∼ 1.3 μmol/L [13]. Therefore, the sub-micromolar levels of DA in plasma might have meaningful biological activities, especially under a repeated-dosing regimen. Taken together, available information indicated that the dose of AGN used in this PK study resulted in potentially pharmacologically relevant plasma pyranocoumarin levels.

It is well known that individual variation in PK is extremely pronounced for xenobiotics that undergo extensive first-pass metabolism and in adults, age and gender make the least apparent contribution to the variance [31]. In our study, we noticed the greatest variation in the *C*_*max*_ and AUC_0–48h_ of DA among the three analytes (D, DA and DOH), as much as 60 fold among individual subjects (Table 2; S1 Table, Individual data). Even for subjects in the same gender, *C*_*max*_ of DA could be more than 20 fold different (S1 Table, Individual data). This could not be accounted for by age or body weight as the linear coefficient of determination (r^2^) was all smaller than 0.1 (Table 2).

Gender did not significantly affect *C*_*max*_ or *AUC*_*0–48h*_ for all 3 analytes (Table 3), although *T*_*max*_ for DOH was significantly longer in women than that in men. This might be attributable to slower gastrointestinal transition rate and possible slower dissolution of vegicaps in women than in men. In our study, we controlled as many variables as possible such that all subjects had normal hepatic and renal function, they took the investigational medicinal product under fasting status and they were offered similar hospital food within the first 24 hours after dosing. In addition, they were all instructed to avoid taking prescription or nonprescription drugs, vitamins, or herbal/dietary supplements from 48 hours preceding study visit until the final blood draw. So the effect of food and associated enzymatic changes should have been well controlled. Since the conversion from DA to DOH was exclusively CYP P450-driven [19], the genetic polymorphism of P450 isoforms might be a major contributor to the PK variation. Since we have collected peripheral blood mononuclear cells (PBMCs), we will test the correlation between plasma DA levels and the CYP P450 genotypes for each individual in the future to test this hypothesis.

In terms of the limitations of our current study, we were cognizant that dietary supplement Cogni.Q was not the ideal form for PK study of D and DA and the small number of subjects in non-Caucasian racial categories. In contrast to established paradigm of drug development in which clinical studies were initiated under full IND approval, we adopted the “Phase 0” mechanism to conduct this PK study. The purpose of the “phase 0” studies is to assist in the “go *vs*. no-go” decision-making process of an agent’s fate earlier in the development process, using human subjects instead of solely relying on animal data. These studies could help confirm end points such as bioavailability and mechanism of action [32]. Knowledge that phytochemicals other than D and DA in AGN extract had minimal effect on their absorption and metabolism in rodent models [17] made our choice of AGN-based dietary supplement scientifically reasonable. In addition to avoiding the tedious IND application, our “serum pharmacochemistry [33]” or “ethnopharmacokinetic [34]” approach allowed us to probe other “bio-available” chemicals in AGN which might exert anti-cancer activities alone or synergistically with D/DA [3]. For example, UHPLC-MS/MS analyses indicated that bergapten, nodakenin, xanthotoxin and isoimperatorin were present in AGN extract and in the plasma of mice treated with AGN extract orally (unpublished data). Xanthotoxin and isoimperatorin were reported to have *in vitro* or *in vivo* anti-cancer activities [2,35]. It will be very interesting to examine their PK parameters using our already collected human samples. These chemicals could work synergistically with D/DA/DOH or even might be developed as separate agents.

## Conclusion

In summary, we conducted a first-in-human single dose PK study of D and DA delivered through Cogni.Q dietary supplement. Our data support the similarity of metabolic fate(s) of D and DA in humans and rodents, with suggestion of a potential slower metabolism of DA in humans than in rodents. The results provide credence to using rodent models to evaluate efficacy and safety data to benefit the clinical translation of AGN phytochemicals on cancer and other diseases and to explore relevant active chemicals and molecular targets.

## Supporting Information

S1 TREND Checklist. (PDF)

S1 Protocol IRB protocol. (PDF)

S1 Table Individual data. (PDF)
