# Supplementary material for: Single Oral Dose Pharmacokinetics of Decursin and Decursinol Angelate in Healthy Adult Men and Women
Source: PLoS One. 2015 Feb 19;10(2):e0114992. doi: 10.1371/journal.pone.0114992 (PMC4335020; doi:10.1371/journal.pone.0114992)
Supplement: S1 Protocol — (PDF) [file pone.0114992.s002.pdf]

TEXAS TECH UNIVERSITY
HEALTH SCIENCES CENTER

INSTITUTIONAL REVIEW BOARD FOR THE PROTECTION OF HUMAN SUBJECTS
FWA # 00006767 AMARILLO IRB #00000097

NOTIFICATION OF INITIAL APPROVAL

January 14, 2013
CORRECTED LETTER

IRB#: A12-3742
STUDY# : Single Oral Dose Pharmacokinetics of Decursin/Decursinol Angelate in Healthy Adult Men and Women (Decursin/Decursinol Angelate)

PRINCIPAL INVESTIGATOR: Jinhui Zhang, Ph.D.
SUBMISSION REFERENCE #: 044883
TYPE OF REVIEW: FULL BOARD

APPROVAL DATE: 01/14/2013
REVIEW PERIOD: 12 Months

RISK ASSIGNMENT: Minimal
EXPIRATION DATE: **12/19/2013**
(based upon date recommended for approval)

# LOCAL SUBJECTS: 30

SPECIFIC INFORMATION PERTAINING TO THIS APPROVAL

IRB members abstaining from discussion/vote due to a potential, or actual, conflict of interest: None.

Documents reviewed and approved include:
1) IRB Application
2) Protocol (iRIS version 1.1) and Appendices E1,2,3,4 and 5
3) Informed Consent dated 12/10/2012 (iRIS version 1.0)
4) Screening Script (iRIS version 1.1)
5) Flyer (iRIS version 1.1)
6) FORMS - Health Questionnaire, Vital Flow Sheet, Physician Check List, Subject Instructions, Master Key, Freezer Log, Study Notes, Study Subject Call Log,
7) LETTERS - Biomedical Sciences Department Letter of Support, FDA Correspondence (Appendice #5),
8) Curriculum Vitae (Dr Jinhui Zhang); Biographical Sketch (Junxuan Lu)
9) IBC License – Dr Junxuan Lu (expires 6/30/2013)

Study Personnel include:
Rakhshanda Rahman, MD, Teresa Baker, MD, Thomas Hale, PhD, Todd E Bell, MD, Joann Urben, Patty Price, BS

Additional Approval May be Required: If parts of this study may take place at non-TTUHSC facilities, respective non-TTUHSC administrative letters of approval must be received *prior to* initiation of research at those settings. Departments/institutions listed in this study recognized as listed in the approved IRB application. If this study requires additional TTUHSC/Committee approvals (example: radiation safety committee review, recombinant DNA biosafety committee review, etc) these approvals must be received *prior to* initiation of the respective research processes.

Approval Period: This approval is for a period of 12 Months. You should receive electronic notification 60 days prior to the expiration of this project's approval. *However, it is your responsibility* to insure that a Continuing Review Submission Form has been submitted by the required time.

Consent Form: The currently approved and stamped consent form must be used when enrolling subjects. You are responsible for maintaining signed consent forms for a period of at least three years after study completion. **NOTE: A HIPAA authorization form is required at the time of obtaining initial consent and whenever the purpose of the study is revised or changed.**

Reporting: The principal investigator must report to the IRB any serious problem, adverse effect, or outcome that occurs with frequency or degree of severity greater than that anticipated. In addition, the principal investigator must report any event or series of events that prompt the temporary or permanent suspension of a research project involving human subjects.

Modifications: Changes or modifications in a research project **must have approval** by the IRB prior to initiation. When modifications are deemed necessary to prevent immediate harm to a subject, changes or modifications must be reported to the IRB within 24 hours.

Study Completion:
If this project is completed within the approval period, you are required to submit a Study Update indicating "Final Closure". The study project is considered completed when:

1) Investigators will not contact subjects for further information related to this project
2) Access to subject health care records are no longer required for information related to this project
3) All IRB requests for information have been completed and no longer require an investigator response
4) A summary report has been completed. This must be attached as a Supporting Document in the Study Update submission.

Clinical Trial Registration:
The sponsor of any clinical trial is encouraged, and in some cases, required to register the study. **ClinicalTrials.gov** is a directory of federally and privately supported research trials designed to test the effect of experimental drugs, devices and procedures for many diseases and conditions. The FDA mandates the registration of clinical trials for efficacy trials of investigational new drugs (INDs) for serious conditions. The International Committee of Medical Journal Editors (ICMJE) requires trial registration as a condition for publication. The National Institutes of Health (NIH) requires registration of all federally funded clinical trials. Registration of a clinical trial is required prior to enrollment of the first subjects in the trial. If this IRB-approved study is a clinical trial which has not been registered by the study sponsor, it may be the Principal Investigator's responsibility to register the trial. For more information on registering a clinical trial, you may consult the following resources: http://prsinfo.clinicaltrials.gov; www.icmje.org/clintrialup.htm ; or www.fda.gov/cder/guidance/4856fnl.htm.

GENERAL INFORMATION

The Texas Tech University Health Sciences Center Institutional Review Boards are duly constituted (fulfilling FDA requirements for diversity), allows only those IRB members who are independent of the investigator and sponsor of the study to vote/provide opinion on the study, has written procedures for initial and continuing review, prepares written minutes of convened meetings, and retains records pertaining to the review and approval process; all in compliance with requirements defined in 21 CFR (Code of Federal Regulations) Parts 50 and 56, and ICH (International Conference on Harmonization) guidance relating to GCP's (Good Clinical Practice).

The Texas Tech University Health Sciences (TTUHSC) Center Policies and Procedures are available for reference on the TTUHSC Human Research Protection Program Website (http://www.ttuhsc.edu/research/hrpo/irb/).

TTUHSC Amarillo Institutional Review Board
1400 Wallace Boulevard
Amarillo, TX 79106
Phone: 806-354-5419

Single Oral Dose Pharmacokinetics of Decursin/Decursinol Angelate in Healthy Adult Men and Women

Principal Investigators:

Jinhui Zhang Ph.D, Department of Biomedical Sciences, School of Pharmacy

Co-Investigators:
Thomas Hale, PhD, Department of Pediatrics, Director of Clinical Research Unit (CRU)

Teresa E. Baker, MD, Department of Obstetrics & Gynecology

Todd Bell, MD, Department of Internal Medicine

Rakhshanda L. Rahman, MD, Department of Surgery

Study Coordinators:
Patty Price, BS, Assistant Director, CRU

Joann Urben, Lead Specialist, CRU

Support Personnel: The Clinical Research Unit (and possibly other staff at TTUHSC) will provide support. All study personnel will be trained and approved by the IRB prior to participation. PRN nurses (RNs or LVNs, trained and approved by the IRB prior to their participation) will cover the 12-hour periods and possibly the subsequent blood draws. If a study nurse does not obtain the subsequent blood draws, a trained phlebotomist or other qualified TTUHSC personnel will perform this task.

Sponsor: School of Pharmacy, TTUHSC

Sites: For the first 12 hours of the study, subjects will be in an unoccupied Northwest Texas Hospital (NWTH) room (the specific room will vary; study staff will coordinate with NWTH when scheduling). After the first 12 hours, subjects will be released and then return to TTUHSC for subsequent blood draws.

Hypothesis:

The *in vivo* metabolism of decursin and decursinol angelate (DA) in humans may be different from that in rodents based on data generated by *in vitro* models. A pilot study on the pharmacokinetics of decursin and DA in healthy subjects will be crucial for selecting relevant models to evaluate the efficacy and safety of corresponding chemicals in a preclinical setting.

Background:

Angelica gigas Nakai (AGN) is a traditional medicinal herb widely used in Asia and in some Western countries as well. Its products are also marketed as food supplements for pain relief, Alzheimer disease and women's health care in the United States. Decursin and its isomer decursinol angelate (DA) are the dominant chemical components in the ethanol extract of the root of AGN. The *in vitro* and *in vivo* anti-cancer, neuro-protective and other biological activities of decursin/DA, as well as AGN extract, have been increasingly reported in the past decade ((1-4), and a comprehensive review recently published by our group (5)). Some food supplements containing AGN extract have been studied for their activities against Alzheimer type dementia and menopausal syndromes in clinical trials (6).

Currently it is believed that decursin/DA contribute to most of the activities of AGN extract reported. In a preclinical setting, we and other groups have convincingly demonstrated that both decursin and DA were rapidly converted to decursinol (DOH) in rats and mice after administration by oral gavage or intraperitoneal (*i.p.*) injection, either in the form of pure chemicals or crude extract (7, 8). Additional experiments by a Korean group and us using *in vivo* and *in vitro* models suggested the liver, instead of gastrointestinal tract and blood, might be the primary organ where the conversion takes place *in vivo* (8). The information raised the hypothesis that DOH could be the real *in vivo* active form, decursin/DA or AGN extract might just serve as "pro-drugs" to deliver DOH *in vivo*. However, the hypothesis was challenged by our recent data, which showed that both decursin and DA were metabolized much slower by human liver microsomes (the primary executors converting decursin/DA to DOH) than mouse and rat liver microsomes ((9) & Appendice E1). This suggested that decursin and DA might dwell longer and at higher concentration in the human body than in rodents. Whether the pharmacokinetics of decursin/DA in human is similar or different from that in rodents could only be addressed by direct testing in human subjects.

A key question for human applicability of *in vivo* efficacy of decursin, DA or AGN extract by extrapolation from observations in rodent models is the pharmacokinetic characteristics of decursin, DA and DOH in humans and whether they are the same or different from rodents. Therefore there is a critical need to perform a pilot pharmacokinetic study in human subjects as the starting point to resolve this issue. If human pharmacokinetics agree with findings in rodents, then regular rodent models will be suitable for preclinical studies of AGN extract, decursin/DA and DOH. If decursin and/or DA do not undergo extensive hydrolysis in humans

during absorption and transport in the circulation, then animal models with blocked or a diminished ability to hydrolyze decursin and/or DA would be essential for efficacy and toxicology evaluations to support an IND application and future clinical trials in humans.

Objective:

This will be the first study of the pharmacokinetics of decursin/DA (in the form of AGN extract) in humans. The primary objective is to obtain information on the pharmacokinetics of decursin/DA, which is crucial for selecting the most relevant animal models to evaluate its efficacy and safety in preclinical stage. A single-dose safety evaluation will be performed concurrently. The secondary objective is to profile the plasma proteome and peripheral mononuclear blood cells (PMBC) transcriptome before and after dosing to obtain insights on potential *in vivo* targets.

Significance:

The anti-cancer activities of AGN extract and decursin/DA against prostate, breast, lung and colon cancer had been studied by us and other groups. Also, their effects on nervous systems such as anti-nociceptive, anti-amnesic activities in several rodent models were also reported. Recently, a phase III efficacy and safety study of INM-176, a brand name of AGN extract manufactured by Whanin Pharmaceutical Company, for the treatment of patients with Alzheimer type dementia (NCT01245530) was completed in Korea. It is well known that prostate cancer is the second leading cause of male cancer death in USA. One out of every six men will develop prostate cancer in their lifetime (10). Aside from the response of this malignancy to chemotherapy, pain in the terminal stages of cancer therapy dramatically decreases the quality of life of cancer patients. Alzheimer's disease is the most common type of dementia; one in eight of older Americans has Alzheimer's disease. Currently, there are 5.2 million people over age 65 (3.4 million women and 1.8 million men) with Alzheimer's in the United States and the life quality of those patients and their families are negatively affected by the disease (11). While we will start with a limited number of heathy individuals (men and women) to initiate the translational study of decursin/DA, the results may generalize to the very large group of older individuals. Thus, the results of these studies could have great significance for public health in a variety of populations.

Study Drug (Information and Risks):

Although neither decursin nor DA has been approved for clinical use in this country, the commercially available food supplement CognIQ™ will be used for this proposed study. CognIQ™ is currently manufactured by Quality of Life Labs (Purchase, NY, USA). It is claimed that CognIQ™ can promote cognitive agility and support healthy brain function, which are consistent with the reported neuro-protective activities of AGN extract (4). The suggested dosage for adults is 4 capsules every day (Appendice E2). According to supplement facts, each capsule

contains patented AGN extract INM176 200mg (equal to 20mg decursin). As mentioned above, a phase III efficacy and safety study of INM-176 for the treatment of patients with Alzheimer type dementia (NCT01245530) was recently completed in Korea (Appendice E3). We confirmed the decursin and DA content in CognIQ™ claimed by the manufacture using our published HPLC-UV method (7). In addition, using state-of-the-art UHPLC-MS/MS (QTRAP5500, ABSCIEX) technology, we compared the chemical fingerprinting profiles of CognIQ™ and AGN extract provided by our collaborators in Korea (Appendice E4). The similarities in the profiles further supported that CognIQ™ contains AGN extract, which we want to study in the pilot trial. Shaw T. Chen, M.D., Ph.D, Deputy Director of Office of Drug Evaluation IV and Leader of Botanical Review Team at the CDER, FDA, has already confirmed with us that no IND application is required if the proposed pilot study will be conducted in normal healthy volunteers (e.g. not cancer patients) and the doses are within the range of dietary supplement use (Appendice E5).

Risk assessment: We do not anticipate the proposed research involves more frequent or greater risks to the subject than the risks ordinarily encountered in daily life. The only risk factor specifically related to the proposed study is a single oral dose of AGN (CognIQ™). As a traditional medicinal herb, AGN has been used for centuries if not longer. Several studies testing the *in vivo* activities of AGN or individual pyranocoumarins (decursin, DA, *etc*) have shown that they were well-tolerated by animals, either by single or repeated dosing, through different administration routes. Recently, we finished two repeat-dose toxicology studies in mice for AGN. In the first study, male C57BL6 mice were exclusively fed with AIN-93M purified diet containing 5000 ppm or 10,000 ppm AGN (roughly equal to human dose 50 and 100 mg/kg per allometric adjustment) for 6 weeks. In a second study, male and female CD-1 mice received daily AGN treatment at dosages of 100, 200 or 300mg/kg levels by *i.p.* injection for 4 weeks, 6 days per week (roughly equal to human dose of 10, 20 and 30mg/kg per allometric adjustment). Body weight, food intake, general behaviors were monitored weekly or daily. After euthanasia, necropsy, plasma biochemistry and histological evaluation of major organs were performed. In both studies, AGN was very well tolerated and we did not find any obvious sign of toxicity or mutagenicity. The data suggested that AGN should not cause significant toxic effect on human beings, especially when only a single dose of less than 10mg/kg is administered orally.

In addition, AGN products are marketed as food supplements in Europe and the United States. CognIQ™, the brand name of AGN extract we propose to use in the study is advertised for promoting cognitive agility and supporting healthy brain function. Other AGN products include D-Cursinol (for back pain and aches), Fast-Acting Joint Formula (for pain-killing), Decursinol-50™ (for minor pain relief), Ache Action™ Decursinol Complex (for temporary relief of minor aches and pains), EstroG-100™ (for premature, perimenopausal, and post-menopausal symptoms). Some of these products have been studied in the clinical setting and no

major side effect was reported (6). Taken together, we do not expect frequent or greater risks related to single dose of AGN in healthy subjects.

Identification/Recruitment of Subjects:

20 subjects will be enrolled in this study. Flyers will be posted at various places in Amarillo/Canyon to recruit subjects. A phone script will be used by study personnel when answering calls from potential subjects for screening.

Inclusion Criteria:

- Health subjects weighing between 50 to 91 kilograms (110 to 200 pounds)
- Subjects 18 to 65 years of age
- Subjects having normal hepatic, renal and bone marrow function as assessed by history, physical and clinical chemistry analysis

Exclusion Criteria:

- Subjects positive for HIV, HBV and HCV
- Subjects with diabetes—due to length of fasting
- Subjects regularly taking any kind of prescription medications
- Subjects taking oral contraception, hormone-containing IUDs, contraception implants, or Depo medroxyprogesterone injections
- Subjects taking any food or herbal supplements containing AGN (*e.g.* CognIQ™, D-Cursinol, Decursinol-50™, Ache Action™, Fast-Acting Joint Formula, EstroG-100™) within 30 days of the study.
- Female subjects that are pregnant, <6 months postpartum, or breastfeeding women.

Study Design:

1. Study personnel (at CRU) will interview candidate subjects over the phone based on Phone Screening Script. Potentially eligible subjects will be scheduled for the screening visit at TTUHSC and their eligibility requirements will be confirmed. During the screening visit, the subject will first sign and date an informed consent document prior to any study procedures. Then the subject will be asked to complete a health history questionnaire. A physical examination will be completed by one of our attending physicians. A "pre-study" blood draw (15 ml) will be collected. Plasma biochemistry will be performed to confirm normal liver, kidney and hematopoietic function of the subject; plasma decursin, DA and DOH levels will also be

analyzed to confirm that subject is not taking any food or herbal supplements containing AGN. The 9 ml blood for hepatic function panel, BUN & creatinine panel and CBC (complete blood count) will be collected in tubes containing SST supplied by Quest and the other 6 ml blood will be collected in tubes containing sodium heparin supplied by the PI.

2. The study staff will set up a time for the eligible subject's return visit to Northwest Texas Hospital (NWTH) where the study is being conducted. The form entitled "Information to Subjects" will be given to the subject at this time. NWTH administration has approved the use of unoccupied rooms at NWTH. The room will vary; study staff will contact NWTH personnel for the room number in preparation for each subject's visit. The subject will remain in the NWTH room with the study nurse for the first 12 hours of the study.

3. For the 48 hours preceding study visit until the final blood draw, the subject will be instructed to avoid consuming alcohol, prescription drugs, and any over-the-counter drugs and food (herbal) supplements. Female subjects will be asked to use a form of birth control other than oral contraception, hormone-containing IUDs, contraception implant, and Depo medroxyprogerone injections throughout the course of the study.

4. Subjects will arrive at NWTH in the early AM on the day after a fast beginning at 10 pm the previous night. Subjects will be encouraged to drink water up until one hour before the drug administration.

5. A urine pregnancy test will be performed for each female subject prior to study drug administration. If the result is positive for pregnancy, the study team will request the subject to quit the study and seek follow-up care with the appropriate health care provider.

6. The study nurse will do the following:

Take vital signs- (temperature, pulse, respirations, and blood pressure)

Place an IV line with saline drip.

Draw an initial sample of blood (18 mL) at pre-dose Hour Zero.

7. The study nurse will then administer four CognIQ™ capsules with 240 mL (8 fluid ounces) of water at time zero. The subject will stay at NWTH in their room for the next 12 hours. If emergency care is necessary, the co-investigators (TTUHSC physicians) will be consulted. All MD co-investigators on this study have privileges at NWTH. If the co-investigator determines emergency care is required, the subject will be referred/admitted to NWTH.

8. No food will be allowed for at least 4 hours post-dose.

9. Water will be allowed as desired, except for one hour after drug administration. After the drug administration, the study nurse will collect blood (6 mL for each draw + 5 mL of waste for each

draw) at hours 0.5, 1, 2, 3, 4, 6, 8 and 12. With the first blood draw (Hour 0), an IV line will be placed to keep the vein open (KVO), avoiding numerous venipunctures. The study nurse will maintain this IV line with a saline drip throughout the 12-hour stay following the study drug administration. Normal saline will be infused at a rate of 10-20 mL/hr. to keep the vein open. Hemodilution will be minimal due to the low infusion rate. Prior to collecting samples, the IV line will be clamped off to stop the flow of NS. Using one of the ports on a 3-way stopcock, 5 mLs of waste will be drawn followed by the sample. The IV line will be unclamped to keep the vein open for the next sample. In the rare case that the IV line becomes blocked and with the patients' permission, the sample will be drawn by IV phlebotomy by our nursing personnel in the alternate arm. The IV line will be removed by the nurse after the 12-hour sample is drawn.

10. Subsequent blood samples on return study visits 24 and 48 hours post dose will be taken by a phlebotomist or nurse at TTHUSC. The amount of blood to be drawn will be 18cc for 24 hours and 6cc for 48 hours. Subjects will be asked to fast beginning at midnight for the 24 hour blood draw.

11. Urine samples (40ml) will be collected within time spans of 0-4, 4-8, 8-12 and 12-24 hours (total volume of urine to be recorded in I&O form). The samples will be stored on dry ice immediately. At the earliest opportunity, the lab personnel will transport the samples from the NWTH room to TTUHSC's -80 freezer.

12. Subjects will receive standardized meals at post-dose hours 4.5 and 9.5. NWTH cafeteria will provide the meal from their in-patient menu. Intake and output will be recorded on the I&O sheet. Vital signs will be taken and recorded pre-dose Hour 0 and post-dose Hours 1, 5, 8, 12, 24 and 48, and at the discretion of research staff.

13. The 9 ml blood drawn at hours 0 and 24 will be collected in tubes containing SST supplied by Quest. These blood samples will be tested for hepatic function panel, BUN & creatinine panel and CBC by Quest. At 30 days post-dose, all participants will be contacted by telephone for toxicity assessments. All toxicities for all consented subjects will be recorded.

14. The rest of blood will be collected in tubes containing sodium heparin supplied by the PI. Samples will be centrifuged at 3000 rpm for > 10 minutes at 4 degrees Celsius. The collected plasma and blood cells from each tube will be placed into separate polypropylene tubes. The samples will be stored on dry ice immediately. At the earliest opportunity, the lab personnel will transport the samples from the NWTH room to TTUHSC's - 80 freezer.

15. The levels of decursin, DA, DOH and other chemicals, as well as their potential metabolites, in plasma/urine samples will be quantitated by LC-MS/MS by the PI and Li Li Ph.D, core facility director of TTUHSC School of Pharmacy (7, 9). Biomarkers related to angiogenesis, inflammation, adipogenesis, *etc* in the blood which could reflect the *in vivo* mechanisms of AGN and decursin/DA (2, 5) will be analyzed at mRNA and/or protein levels by the PI.

Data management:

**Name of form**	**Description**	**Completed by**	**When completed**	**Where it will be completed/stored**
**Master Key**	to track participants, his/her subject number, appointment dates	approved study support staff	when the informed consent is signed	in the CRU in a locked cabinet
**Information to Subjects**	for the subject to take home as reference for future appointments and what food and drinks to avoid	study nurse	After the Informed Consent Document visit	given to subjects
**Physician Checklist**	form the physician will use to confirm eligibility and record health status of the subject	study physician	completed when the study physician completes the history and physical examination	completed at TTUHSC and kept in each subject's file, locked in the CRU cabinet
**Health History Questionnaire**	used to record the health status/history of the subject	study nurse	completed at the same visit as the ICD and/or physical – completed 1 time	completed at TTUHSC and kept in each subject's file, locked in the CRU cabinet
**Flowsheet**	record vital signs and nurses notes	study nurse	throughout the 12-hour stay at NWTH	completed at NWTH and kept in each subject's file, locked in the CRU cabinet
**Phone Screening**	for use when potential subjects	TTUHSC personnel (all	throughout the	TTUHSC

**Script**	call to get information about the study	approved study members) answering the phone	study	
**I&O (together with Flowsheet)**	to record the intake and output of subjects during the 12 hour stay at NWTH	study nurse	throughout the 12-hour stay at NWTH	completed at NWTH, kept in each subject's file, locked in the CRU cabinet
**Call Log**	to record telephone conversations for tracking purposes (# of people who call with interest in the study, description of enrolled subjects calling with questions/ problems) . The information recorded will not be used for any other purpose beyond this research study.	TTUHSC personnel (all approved study members) answering the phone	throughout the study	TTUHSC
**Freezer Log**	to record the temperature of the freezer and to log sample storage/shipping	Lab personnel	throughout the study	PI's lab
**Record of adverse effects (together with Flowsheet)**	Recorded on the nursing notes at the time, reported to IRB also	Study nurse, physician	throughout the study	At TTUHSC after the 12-hour period at NWTH

Subject Incentive Payments:

Each subject will receive a total of $400 for completing the study in full. For completing the 12-hour period, the subject will receive $200. For each of the blood draws at hours 24 and 48, the subject will receive $100 for each draw. Payments will be via check through the TTUHSC direct pay system. If the patient cannot complete the study after the initial blood work they will receive $50 for their time.

Safety Monitoring:

The risk related to the administration of CognIQ^{TM} is minimal. To minimize the possibility of minor and reversible side effects, presumably due to psychological anxiety, we will only recruit healthy participants (normal liver and kidney functions, *etc*) and exclude subjects with diabetes and pregnant, perinatal and breastfeeding women. In addition, we will closely monitor any possible side effect during the whole study section. Full-time medical supervision is to be provided by personnel qualified to provide emergency medical care during confinement. A urine pregnancy test is to be performed for each female subject prior to drug administration. Vital signs (blood pressure and pulse) are to be monitored at pre-dose Hour 0 and post-dose Hours 1, 5, 8, 12, 24, and at the discretion of the research staff. If emergency care is necessary, one of the co-investigators (TTUHSC physicians) will be consulted. All co-investigators on this study have privileges at NWTH. If the co-investigator determines emergency care is required, the subject will be referred/admitted to NWTH. If a subject experiences a significant side effect due to the drug, the subject may be required to remain in the hospital for a period of up to 3 days, or until the symptoms clear. Study personnel and the on-call physician will confer with Dr. Zhang to determine whether or not the event was related to the study. Study personnel will also document and report the adverse effects including but not limited to the following symptoms during the study to the IRB:

· Infection
· Fever
· Pain
· Abdominal pain
· Asthenia
· Hot flashes
· Skin rashes
· Dry mouth
· Palpitation
· Hypertension
· Hypotension

· Nausea
· Constipation
· Diarrhea
· Anorexia
· Vomiting
· Dysphagia
· Appetite increase
· Dyspepsia
· Blurry vision
· Sore throat
· Urinary frequency
· Headache (including migraine)

Statistical Analysis:

The primary objective of the proposed study is to investigate whether human beings, as a biological species, will metabolize decursin and DA differently from rodents. All the chemicals we are interested in including decursin, DA, DOH do not naturally exist in human body, so only one treatment group (single oral dose based on the recommend dose by the manufacture) will be included in this study. Under this scenario, the proposed study should be considered as a "qualitative" experiment rather than a "quantitative" experiment. No sample size justification was provided, because it is a pilot study. The pharmacokinetic program WinNonlin will be used to calculate the pharmacokinetic parameters such as maximum concentration (C_{max}) and area under the curve (AUC) of decursin, DA and DOH. Box-plot graphics will be also used to describe in more detail differences in C_{max} and AUC. If a considerable amount of decursin and/or DA is detected in the plasma of all or some of subjects, we may plan additional trials which will recruit more subjects to investigate the factors affecting pharmacokinetic behavior of corresponding chemicals such as gender, race, CYP genotypes, *etc.*

Protection of Confidential Information:

Consent procedures will take place in the CRU or a private room at TTUHSC. Only research team members will be authorized to consent subjects. All PHI and research data will be maintained in a secure location in an office that requires a key for entrance. Study documents with identifiable data (such as the consent forms) will be stored in locked files. Only the research support staff, the Principal Investigators, and the co-investigators will have access to study records. Study-related data will be maintained on password-protected computers. The study data or test results will be part of a research record separate from the subject's medical record.

Blood samples drawn will be de-identified prior to being analyzed. They will be marked only with the subject's assigned study number (the master key maintained in a locked cabinet at

the CRU will be the only means of linking these study numbers to individuals) , the date and time the sample was obtained, and the initials of the phlebotomist/nurse drawing the sample.

Reference

1. Son SH, Park KK, Park SK, Kim YC, Kim YS, Lee SK, Chung WY. Decursin and Decursinol from Angelica gigas Inhibit the Lung Metastasis of Murine Colon Carcinoma. *Phytother Res* 2010. Available at http://www.ncbi.nlm.nih.gov/pubmed/21170925.
2. Lee HJ, Lee EO, Lee JH, Lee KS, Kim KH, Kim SH, Lu J. In vivo anti-cancer activity of Korean Angelica gigas and its major pyranocoumarin decursin. *Am J Chin Med* 2009;37:127-142. Available at http://www.ncbi.nlm.nih.gov/pubmed/19222117.
3. Wang L, Zhang Y, Zhang J, Nkata K, Quealy E, Li L, Lee HJ, Jeong SJ, Liao JD, Jiang C, Kim SH, Lü J. Korean Angelica gigas Nakai (AGN) and Oriental herbal cocktail ka-mi-kae-kyuk-tang (KMKKT) inhibit prostate carcinogenesis in TRAMP model. . *Proc AACR* 2011:Abstract #5581.
4. Kang SY, Lee KY, Park MJ, Kim YC, Markelonis GJ, Oh TH. Decursin from Angelica gigas mitigates amnesia induced by scopolamine in mice. *Neurobiol Learn Mem* 2003;79:11-18. Available at <Go to ISI>://000180347400002.
5. Zhang J, Li L, Jiang C, Xing C, Kim SH, Lu J. Anti-cancer and Other Bioactivities of Korean Angelica gigas Nakai (AGN) and Its Major Pyranocoumarin Compounds. *Anticancer Agents Med Chem* 2012. Available at http://www.ncbi.nlm.nih.gov/pubmed/22583405.
6. Chang A, Kwak BY, Yi K, Kim JS. The effect of herbal extract (EstroG-100) on pre-, peri- and post-menopausal women: a randomized double-blind, placebo-controlled study. *Phytotherapy research : PTR* 2012;26:510-516. Available at http://www.ncbi.nlm.nih.gov/pubmed/21887807.
7. Li L, Zhang J, Shaik AA, Zhang Y, Wang L, Xing C, Kim SH, Lu J. Quantitative determination of decursin, decursinol angelate, and decursinol in mouse plasma and tumor tissue using liquid-liquid extraction and HPLC. *Planta Med* 2012;78:252-259. Available at http://www.ncbi.nlm.nih.gov/pubmed/22116603.
8. Park HS, Kim B, Oh JH, Kim YC, Lee YJ. First-pass Metabolism of Decursin, a Bioactive Compound of Angelica gigas, in Rats. *Planta Med* 2012. Available at http://www.ncbi.nlm.nih.gov/pubmed/22573368.
9. Li L, Zhang J, Xing CG, Jiang C, Kim SH, Zhang R, Lü J. In vitro Metabolism Studies of Herbal Compound Decursin and Decursinol angelate in rodent and Human Liver Microsomes *60th ASMS Conference on Mass Spectrometry and Allied Topics* 2012.
10. Siegel R, Naishadham D, Jemal A. Cancer statistics, 2012. *CA Cancer J Clin* 2012;62:10-29. Available at http://www.ncbi.nlm.nih.gov/pubmed/22237781.
11. 2012 Alzheimer's disease facts and figures. *Alzheimers Dement* 2012;8:131-168. Available at http://www.ncbi.nlm.nih.gov/pubmed/22404854.
