# Supplementary material for: Single Oral Dose Pharmacokinetics of Decursin and Decursinol Angelate in Healthy Adult Men and Women
Source: PLoS One. 2015 Feb 19;10(2):e0114992. doi: 10.1371/journal.pone.0114992 (PMC4335020; doi:10.1371/journal.pone.0114992)
Supplement: S1 Table — (PDF) [file pone.0114992.s003.pdf]

Supplemental Table 1

Subject #	S4	S6	S17	S1	S2	S7	S9	S12
Gender	F	F	F	F	F	F	F	F
Age, years	51	40	31	40	32	46	31	39
Body Weight (lb)	170	120	147	115	194	180	158	169
D (mg/kg)	1.55	2.19	1.79	2.29	1.36	1.46	1.66	1.56
DA (mg/kg)	0.99	1.41	1.15	1.47	0.87	0.94	1.07	1.00
T_{max} D, h	2	3	2	1	2	4	1	2
T_{max} DA, h	2	3	2	2	3	4	1	2
T_{max} DOH, h	6	4	2	3	4	6	3	4
C_{max} D, nM	13.2	13.4	9.8	4.7	2.0	3.6	2.4	1.3
C_{max} DA, nM	100.2	84.3	103.5	23.4	13.4	74.3	13.6	16.8
C_{max} DOH, nM	2681	2771	3742	2379	2388	3969	2114	2593
$AUC_{0\text{-}48h}$ D	119.0	94.0	53.9	45.0	14.8	23.3	20.6	24.8
$AUC_{0\text{-}48h}$ DA	1059.4	696.4	593.0	190.4	97.5	432.2	80.8	150.5
$AUC_{0\text{-}48h}$ DOH	63735	37474	46744	17573	25032	51798	17727	29072
Terminal $t_{1/2}$, D, h	10.8	12.8	22.7	"173	16.9	24.7	24	15.7
Terminal $t_{1/2}$, DA, h	17.7	16.2	24.2	"20.2	38.6	17.4	19.1	40.2
Terminal $t_{1/2}$, DOH, h	11.1	7.2	10.2	"9.9	9.2	6.5	7.7	7.9
				outlier				

S18	S19	S3	S5	S8	S13	S10	S11	S14	S15
F	F	M	M	M	M	M	M	M	M
22	24	33	32	24	34	58	24	30	24
180	130	170	161	160	192	185	178	200	177
1.46	2.02	1.55	1.63	1.64	1.37	1.42	1.48	1.31	1.49
0.94	1.30	0.99	1.05	1.05	0.88	0.91	0.95	0.84	0.95
4	3	4	1	3	0.5	0.5	1	1	1
4	4	6	1	3	1	2	1	1	1
3	6	6	2	3	2	2	2	2	1
1.7	1.7	1.6	2.0	9.6	1.3	5.7	2.5	8.6	16.8
3.0	6.9	27.2	21.4	183.1	11.2	7.0	29.7	24.4	196.3
1156	2271	1558	1526	2529	2415	1608	3420	3337	4018
25.8	35.7	9.8	34.3	79.2	13.5	46.6	14.7	21.5	23.8
48.1	87.7	141.5	293.5	1601.7	105.7	115.3	168.1	96.4	512.2
16491	21378	8311	19224	30352	26086	17410	37280	25671	26061
"138.5	8.5	"69.6	22.8	9.9	25.8	13.4	20.2	4.5	26.5
"55.7	16	"31.4	12	16.7	12.7	13.3	14.2	12.6	18.7
"16	6.6	"5.2	7.7	5.5	6	8.8	5	5.3	4.5
outlier		outlier							

S16	S20	Subject #	Women Mean, F	SD, F	Men Mean, M	SD, M	Grand Mean	Grand SD
M	M	Gender	n=10		n=10		n=20	
21	33	Age, years	35.6	9.3	31.3	10.5	33.5	
175	200	Body Weight (lb)	156	27	180	14	168	24
1.50	1.31	D (mg/kg)	1.73	0.33	1.47	0.12	1.60	0.27
0.96	0.84	DA (mg/kg)	1.11	0.21	0.94	0.08	1.03	0.18
3	2	T_{max} D, h	2.4	1.1	1.7	1.2	2.1	1.2
3	2	T_{max} DA, h	2.7	1.1	2.1	1.6	2.4	1.4
2	2	T_{max} DOH, h	4.1	1.4	2.4	1.3	3.3	1.6
2.8	1.3	C_{max} D, nM	5.37	4.87	5.22	5.07	5.30	4.84
4.3	17.2	C_{max} DA, nM	43.94	41.25	52.18	73.04	48.06	57.89
1798	1333	C_{max} DOH, nM	2606	798	2354	950	2480	864
22.2	19.1	$AUC_{0\text{-}48h}$ D	45.7	34.6	28.5	20.8	37.1	29.2
68.4	169.5	$AUC_{0\text{-}48h}$ DA	343.6	341.6	327.2	466.5	335.4	398.0
16316	17854	$AUC_{0\text{-}48h}$ DOH	32702	16546	22457	8256	27579	13769
"9.4	18.4	Terminal $t_{1/2}$, D, h	17.0	6.2	17.7	7.8	17.4	6.8
"190.2	18.8	Terminal $t_{1/2}$, DA, h	23.7	10.0	14.9	2.8	19.3	8.5
"15	9.6	Terminal $t_{1/2}$, DOH, h	8.3	1.7	6.6	1.9	7.4	2.0
outlier								
